# Supplementary material for: A brainstem–hypothalamus neuronal circuit reduces feeding upon heat exposure
Source: Nature. 2024 Mar 27;628(8009):826–34. doi: 10.1038/s41586-024-07232-3 (PMC11041654; doi:10.1038/s41586-024-07232-3)
Supplement: Supplementary file 1 — Reporting Summary [file 41586_2024_7232_MOESM1_ESM.pdf]

nature portfolio

Corresponding author(s): Dr. Tibor Harkany

Last updated by author(s): Dec 5, 2023

Reporting Summary

Nature Portfolio wishes to improve the reproducibility of the work that we publish. This form provides structure for consistency and transparency in reporting. For further information on Nature Portfolio policies, see our Editorial Policies and the Editorial Policy Checklist.

Statistics

For all statistical analyses, confirm that the following items are present in the figure legend, table legend, main text, or Methods section.

n/a	Confirmed	
☐	☒	The exact sample size (*n*) for each experimental group/condition, given as a discrete number and unit of measurement
☐	☒	A statement on whether measurements were taken from distinct samples or whether the same sample was measured repeatedly
☐	☒	The statistical test(s) used AND whether they are one- or two-sided<br>*Only common tests should be described solely by name; describe more complex techniques in the Methods section.*
☒	☐	A description of all covariates tested
☐	☒	A description of any assumptions or corrections, such as tests of normality and adjustment for multiple comparisons
☐	☒	A full description of the statistical parameters including central tendency (e.g. means) or other basic estimates (e.g. regression coefficient) AND variation (e.g. standard deviation) or associated estimates of uncertainty (e.g. confidence intervals)
☐	☒	For null hypothesis testing, the test statistic (e.g. *F*, *t*, *r*) with confidence intervals, effect sizes, degrees of freedom and *P* value noted<br>*Give P values as exact values whenever suitable.*
☒	☐	For Bayesian analysis, information on the choice of priors and Markov chain Monte Carlo settings
☒	☐	For hierarchical and complex designs, identification of the appropriate level for tests and full reporting of outcomes
☒	☐	Estimates of effect sizes (e.g. Cohen's *d*, Pearson's *r*), indicating how they were calculated

Our web collection on statistics for biologists contains articles on many of the points above.

Software and code

Policy information about availability of computer code

Data collection	1) Electrophysiological data were collected using Clampex 10.7 (Molecular Devices); Clampfit 10.7, and PatchMaster Next (Heka).<br>2) Confocal image acquisition was done using the Zen Black Edition (Zeiss) software package.<br>3) Calcium imaging data were collected using the Visiview Software (version 3.0.3.0; Visitron Systems).<br>4) qPCR data were collected by the Bio-Rad CFX Manager software (version 3.1, Bio-Rad).<br>5) Behavioral data were captured with EthoVision XT14 (Noldus).<br>6) Primers were designed using Primer3web, version 4.1.0.
Data analysis	Images were analyzed using Imaris x64 9.0.2 (Bitplane) and Fiji 1.52e (GNU General Public Licence, https://imagej.net/Fiji).<br>Electrophysiology data (EPSCs) were analyzed by the Mini Analisys Program (version 6.0; Synaptosoft).<br>Data were processed in Microsot Excel (version 16.79.2), and statistically analyzed by GraphPad Prism 8.0.2 (GraphPad Software Inc.).

For manuscripts utilizing custom algorithms or software that are central to the research but not yet described in published literature, software must be made available to editors and reviewers. We strongly encourage code deposition in a community repository (e.g. GitHub). See the Nature Portfolio guidelines for submitting code & software for further information.

Data

Policy information about availability of data

All manuscripts must include a data availability statement. This statement should provide the following information, where applicable:

- Accession codes, unique identifiers, or web links for publicly available datasets
- A description of any restrictions on data availability
- For clinical datasets or third party data, please ensure that the statement adheres to our policy

No data were included that shall be placed in a public repository. Single-cell RNA-seq data were reprocessed from public libraries as identified. All individual data were presented as single points, with the corresponding "raw data" and statistical analyses made available in the Source Data File that is part of this submission.

Human research participants

Policy information about studies involving human research participants and Sex and Gender in Research.

Reporting on sex and gender	n/a
Population characteristics	n/a
Recruitment	n/a
Ethics oversight	n/a

Note that full information on the approval of the study protocol must also be provided in the manuscript.

Field-specific reporting

Please select the one below that is the best fit for your research. If you are not sure, read the appropriate sections before making your selection.

☒ Life sciences ☐ Behavioural & social sciences ☐ Ecological, evolutionary & environmental sciences

For a reference copy of the document with all sections, see nature.com/documents/nr-reporting-summary-flat.pdf

Life sciences study design

All studies must disclose on these points even when the disclosure is negative.

Sample size	The sample size has been chosen as specified in previous publications PMID: 24121436; PMID: 32917598.
Data exclusions	No data were excluded from the analysis.
Replication	The experiments reported here were minimally performed in duplicates (biological repeats in two (or more) independent experimental settings). All attempts of replication were successful.
Randomization	Experimental animals used in this study were not randomized. This is mainly because of their complex genetic features and since representatives of all experimental groups were tested in parallel to minimize bias (e.g., in multi-chamber phenotyper systems).
Blinding	Experimenters were not blinded to the experimental conditions, because experimental manipulations were measured in automated systems and run as consecutive experiments with the re-use of the same animals (i.e. baseline at start, experimental conditions thereafter. Moreover, the complex genetics of the animals and the relatively low numbers of the experimental subjects necessitated clear group assignments.

Reporting for specific materials, systems and methods

We require information from authors about some types of materials, experimental systems and methods used in many studies. Here, indicate whether each material, system or method listed is relevant to your study. If you are not sure if a list item applies to your research, read the appropriate section before selecting a response.

Materials & experimental systems

n/a	Involved in the study
☐	☒ Antibodies
☒	☐ Eukaryotic cell lines
☒	☐ Palaeontology and archaeology
☐	☒ Animals and other organisms
☒	☐ Clinical data
☒	☐ Dual use research of concern

Methods

n/a	Involved in the study
☒	☐ ChIP-seq
☒	☐ Flow cytometry
☒	☐ MRI-based neuroimaging

Antibodies

Antibodies used

Primary antibodies:
guinea-pig anti-cFOS (1:1,000; Synaptic Systems, #226005)
rabbit anti-cFOS (1:2,000; Synaptic Systems, #226003)
rabbit anti-DsRed (1:200; Clontech/Takara, #632496)
rabbit anti-RFP, biotin-conjugated (1:1000; Rockland, #600-406-379)
chicken anti-RFP (1:500; Rockland, #600-901-379)
goat anti-GFP (1:200; Abcam, #ab6662)
goat anti-mCherry (1:500; Antibodies Online, #ABIN1440058)
guinea-pig anti-GluA1 (1:100; Alomone labs, #AGP-009)
rabbit anti-GluA2 (1:100; Alomone labs, #AGC-005)
mouse anti-nestin (clone rat-401; 1:500; Millipore, #MAB353)
chicken anti-NeuN (1:500; Millipore, #ABN91)
rabbit anti-p44/42 MAPK (pERK1/2) (Thr202/Tyr204; 1:200; Cell Signaling Technology, #9101S)
rabbit anti-tyrosine hydroxylase (1:500; Millipore, #AB152)
rabbit anti-VGLUT2 (1:200; Synaptic Systems, #135403) or as gift of M. Watanabe (see Ref. 59 for quality controls)
chicken anti-vimentin (1:500; Synaptic Systems, #172006)
goat anti-VEGFA (1:100; R&D Systems, #AF-493-NA)

Secondary antibodies:
Alexa Fluor 488 donkey anti-rabbit IgG (1:2,000; Invitrogen, #AB21206)
Alexa Fluor 488-conjugated AffiniPure donkey anti-guinea pig IgG (1:300; Jackson ImmunoResearch, #706-545-148)
Alexa Fluor 488-conjugated AffiniPure donkey anti-mouse IgG (1:300; Jackson Immu-noResearch, #715-545-151)
Alexa Fluor 647-conjugated AffiniPure donkey anti-rabbit IgG (1:300; Jackson ImmunoResearch, #711-605-152)
Cy2-conjugated AffiniPure donkey anti-goat IgG (1:300; Jackson ImmunoResearch, #705-225-147)
Cy2-conjugated AffiniPure donkey anti-rabbit IgG (1:300; Jackson ImmunoResearch, #711-225-152)
Cy3-conjugated AffiniPure don-key anti-chicken IgG (1:300; Jackson ImmunoResearch, #703-165-155)
Cy3-conjugated Affin-iPure donkey anti-guinea pig IgG (1:300; Jackson ImmunoResearch, #706-165-148)
Cy3-conjugated AffiniPure donkey anti-rabbit IgG (1:300; Jackson ImmunoResearch, #711-165-152)
Cy5-conjugated AffiniPure donkey anti-chicken IgG (1:300; Jackson ImmunoResearch, #703-175-155)
Cy5-conjugated AffiniPure donkey anti-guinea pig IgG (1:300; Jackson Immu-noResearch, #706-175-148)
Cy5-conjugated streptavidin (1:200; Jackson ImmunoResearch, #016-170-084)

Hoechst 33,342 (1:10,000; Sigma Aldrich) was used as nuclear counterstain.

Validation

We refer to the RRID portal for antibodies (https://scicrunch.org/resources/) for validation of the immunoreagents described here. For each antibody, the RRID repository number provides exhaustive data on the host species, titers, links to manufacturer websites, and relevant citations.

Primary antibodies, as specified in the manuscript:
guinea-pig anti-cFOS (Synaptic Systems Cat# 226 005, RRID:AB_2800522)
rabbit anti-cFOS (Synaptic Systems Cat# 226 003, RRID:AB_2231974)
rabbit anti-DsRed (Takara Bio Cat# 632496, RRID:AB_10013483)
rabbit anti-RFP. biotin-conjugated (Rockland, #600-406-379; RRID:AB_828390)
chicken anti-RFP (Rockland, #600-901-379, RRID:AB_10704808)
goat anti-mCherry (Antibodies Online, #ABIN1440058. Used as recommeded by the manufacturer: https://www.antibodies-online.com/antibody/1440058/anti-mCherry+Fluorescent+Protein+antibody/. Recently used in https://doi.org/10.1101/2023.02.03.527010).
goat anti-GFP (Abcam Cat# ab6662, RRID:AB_305635)
guinea-pig anti-GluA1 (Alomone Labs Cat# AGP-009, RRID:AB_2340961)
rabbit anti-GluA2 (Alomone Labs Cat# AGC-005, RRID:AB_2039881)
mouse anti-nestin (Millipore Cat# MAB353, RRID:AB_94911)
chicken anti-NeuN (Millipore Cat# ABN91, RRID:AB_11205760)
rabbit anti-p44/42 MAPK (pERK1/2), used as recommended by the manufacturer in:https://www.cellsignal.de/products/primary-antibodies/phospho-p44-42-mapk-erk1-2-thr202-tyr204-antibody/9101?gclid=Cj0KCQjwuuKXBhCRARIsAC-gM0iy9SRAUFwQ4SrB439mSrmAl1yb6WrECKWoYxjDx5mjlumgoKvFk3saAh_dEALw_wcB&gclsrc=aw.ds. In here, 10 different publications are cited that have validated the antibody.
rabbit anti-tyrosine hydroxylase (Millipore Cat# AB152, RRID:AB_390204)
rabbit anti-VGLUT2 (Synaptic Systems Cat# 135 403, RRID:AB_887883) or as gift of M. Watanabe as described in: https://doi.org/10.1046/j.1460-9568.2003.02698.x

chicken anti-vimentin (Synaptic Systems Cat# 172 006, RRID:AB_2800525)
goat anti-VEGFA (1:100; R&D Systems, #AF-493-NA) Used as recommended by manufacturer in https://www.rndsystems.com/products/mouse-vegf-164-antibody_af-493-na?utm_source=antibodypedia&utm_medium=referral&utm_campaign=product&utm_term=primaryantibodies

Animals and other research organisms

Policy information about studies involving animals; ARRIVE guidelines recommended for reporting animal research, and Sex and Gender in Research

Laboratory animals	For behavioral, electrophysiological, and biochemical experiments only male mice aged P60-P100 days were used. The following mice (wild-type and transgenic) and their crosses as specified were included in our study:<br>C57Bl6/J (as wild-type, local breeding)<br>C57Bl6/N (as wild-type, local breeding)<br>Raxtm1.1(cre/ERT2)Sbls/J (The Jackson Laboratory #025521)<br>B6.Cg-Gt(ROSA)26Sortm14(CAG-tdTomato)Hze/J (The Jackson Laboratory #007914)<br>B6;129P2-Mapttm2Arbr/J (The Jackson Laboratory #021162)<br>B6;129S6-Polr2aTn(pb-CAG-GCaMP5g,-tdTomato)Tvrd/J (The Jackson Laboratory #024477)<br>B6;129S-Slc17a6tm1.1(flpo)Hze/J (The Jackson Laboratory #030212)<br><br>Animals were group housed at standard laboratory conditions unless specified otherwise. Behavioral experiments were performed after sufficiently-long periods of acclimatization to new environments and single housing, as specified in the Methods section.<br><br>For primary cultures of tanycytes, both male and female Wistar rats (at P10) were used.<br>To extract cerebrospinal fluid, male Wistar rats at P60 (Janvier) were used.
Wild animals	No wild animals were used in the study.
Reporting on sex	Males and females were used in this study. Sex was specified in each relevant figure/figure panel.
Field-collected samples	No field collected samples were used in the study
Ethics oversight	Experimental procedures on mice conformed to the 2010/63/EU directive and were approved by the Austrian Ministry of Education, Science and Research (66.009/0145-WF/II/3b/2014 and 66.009/0277-WF/V3b/2017). All procedures were planned to reduce suffering, as well as animal numbers.

Note that full information on the approval of the study protocol must also be provided in the manuscript.
